# Experimental Evidence of Ciguatoxin Accumulation and Depuration in Carnivorous Lionfish

**DOI:** 10.3390/toxins13080564

**Published:** 2021-08-11

**Authors:** Isabel do Prado Leite, Khalil Sdiri, Angus Taylor, Jérôme Viallon, Hela Ben Gharbia, Luiz Laureno Mafra Júnior, Peter Swarzenski, François Oberhaensli, Hélène Taiana Darius, Mireille Chinain, Marie-Yasmine Dechraoui Bottein

**Affiliations:** 1 Center for Marine Studies, Federal University of Paraná. Av. Beira-mar, s/n, Pontal do Paraná P.O. Box 61, Brazil; luiz.mafra@ufpr.br; 2 Université Côte d’Azur, CNRS, ECOSEAS, UMR7035, Parc Valrose, CEDEX 2, 06103 Nice, France; sdiri.khalil06@gmail.com; 3 Environment Laboratories, Department of Nuclear Science and Application, International Atomic Energy Agency, 4 Quai Antoine 1er, 98000 Monaco, Monaco; angustaylor93@gmail.com (A.T.); P.Swarzenski@iaea.org (P.S.); F.R.Oberhaensli@iaea.org (F.O.); 4 Laboratory of Marine Biotoxins, Institut Louis Malardé, UMR EIO (IFREMER, IRD, ILM, UPF), P.O. Box 30 Papeete, Tahiti, French Polynesia; jviallon@ilm.pf (J.V.); tdarius@ilm.pf (H.T.D.); mchinain@ilm.pf (M.C.); 5 MMS Laboratory (EA 2160), Sciences and Techniques Faculty, Le Mans University, Avenue Olivier Messiaen, 72085 Le Mans, France; Hela.Ben_Gharbia@univ-lemans.fr; 6 Visiting Scientist Ifremer, Laboratoire Phycotoxines, Rue de I’lle d’Yeu, 44311 Nantes, France

**Keywords:** ciguatoxins, experimental exposure, lionfish, trophic transfer, toxin accumulation, ciguatera poisoning

## Abstract

Ciguatera poisoning is a food intoxication associated with the consumption of fish or shellfish contaminated, through trophic transfer, with ciguatoxins (CTXs). In this study, we developed an experimental model to assess the trophic transfer of CTXs from herbivorous parrotfish, *Chlorurus microrhinos*, to carnivorous lionfish, *Pterois volitans*. During a 6-week period, juvenile lionfish were fed naturally contaminated parrotfish fillets at a daily dose of 0.11 or 0.035 ng CTX3C equiv. g^−1^, as measured by the radioligand-receptor binding assay (r-RBA) or neuroblastoma cell-based assay (CBA-N2a), respectively. During an additional 6-week depuration period, the remaining fish were fed a CTX-free diet. Using r-RBA, no CTXs were detectable in muscular tissues, whereas CTXs were measured in the livers of two out of nine fish sampled during exposure, and in four out of eight fish sampled during depuration. Timepoint pooled liver samples, as analyzed by CBA-N2a, confirmed the accumulation of CTXs in liver tissues, reaching 0.89 ng CTX3C equiv. g^−1^ after 41 days of exposure, followed by slow toxin elimination, with 0.37 ng CTX3C equiv. g^−1^ measured after the 6-week depuration. These preliminary results, which need to be pursued in adult lionfish, strengthen our knowledge on CTX transfer and kinetics along the food web.

## 1. Introduction

*Gambierdiscus* spp. are benthic dinoflagellates that may produce lipophilic ciguatoxins (CTXs), as well as other bioactive compounds, including maitotoxins (MTXs), gambierone, gambieroxides, gambierol, and gambieric acid [1,2,3,4,5,6]. Ciguatoxins are considered the primary cause of Ciguatera poisoning (CP) in humans, affecting up to 500,000 fish consumers every year, including rare lethal cases [7,8,9,10]. Affected people may experience temporary and/or persistent/recurrent neurological effects resulting from CTXs binding to voltage-gated sodium channels in excitable tissues [11,12,13,14,15]. Ciguatera symptoms include the tingling of body extremities and/or cold allodynia and, in more severe cases, cardiovascular and respiratory insufficiency leading to coma and death [12]. Chronic neurological manifestations may persist for weeks, months, and in severe cases, years after the first exposure to CTXs [8,16,17]. Symptoms may vary geographically according to the dominant toxin profile in each region. Gastrointestinal disorders such as nausea, vomiting, abdominal pain, and diarrhea are predominantly manifested in the Caribbean region, where fish are contaminated by the so-called Caribbean ciguatoxins (C-CTXs). In contrast, in the Pacific and Indian Oceans, neurotoxic symptoms may predominate following the consumption of fish containing P-CTXs and I-CTXs, respectively. Neurotoxic symptoms may include hallucinations mostly among victims from the Indian Ocean [8].

The effects of CTXs on marine fauna are less documented. In fish, adverse effects from exposure to CTX have been observed in fresh/brackish water species such as blueheads, *Thalassoma bifasciatum* [18], juveniles of *Oreochromis* sp. [19], larvae and embryos of the genus *Oryzias* [20,21,22,23,24], and adult *Mugil cephalus* [25]. Ciguatoxins found in the brain, liver, and muscles of stranded *Monachus schauinslandi* monk seals [26] suggest that marine mammals may also suffer from CTX exposure, and that these compounds persist within the complex marine food webs. However, marine species experimentally fed with CTX (*Naso brevirostris* [27] and *Epinephelus coioides* [28]) did not seem sensitive to the effects of CTX. The fish resistance mechanism to CTX is still unknown.

Following the ingestion of *Gambierdiscus* cells by herbivorous and omnivorous organisms, CTX-like toxins are biotransformed and transmitted along the trophic food webs to top-chain carnivores, including fish, such as groupers and snappers, and marine mammals [28,29,30,31]. More than 400 species of fish are suspected as potential vectors of CP to humans [32]. Coral reef fish known to accumulate CTXs include barracuda, grouper, snapper, moray eel, parrotfish, trevally, and wrasse [33,34]. In general, higher-level carnivorous fish exhibit greater toxin concentrations than smaller fish and herbivores [35,36,37], but this is not always the case [38,39,40,41,42]. The complex processes and kinetics involving CTX accumulation, elimination, and trophic transfer to carnivores are still poorly understood and limited to field observations [42] and scarce laboratory studies [28].

Lionfish, *Pterois volitans* (Scorpaenidae), are carnivorous fish with venom-containing spines [43]. They are native to the Indo-Pacific region and were mostly likely introduced through aquarium releases into the Atlantic Ocean, becoming invasive to the Caribbean Sea and the Gulf of Mexico [44,45]. Due to the absence of natural predators for lionfish in reef ecosystems where they have been recently introduced, their use as a fishery resource has been stimulated to reduce the adverse ecological impacts on reef communities [46,47]. However, as with many other species, lionfish may accumulate CTXs and reach toxin concentrations above safety levels for human consumption as recommended by the U.S. Food and Drug Administration (0.1 ng C-CTX1 equiv. g^−1^, or 0.01 ng CTX1B equiv. g^−1^) [48]. Concentrations of CTXs in lionfish vary geographically, primarily depending on the local abundance and composition/dominance of *Gambierdiscus* species. In the Caribbean Ciguatera-endemic region, for instance, CTX accumulation in lionfish muscles can be highly frequent (≥40–50%), and reach levels up to 0.3 ng C-CTX1 equiv. g^−1^ [46,49]. This suggests that *P. volitans* could become a common vector for CP in that region, where other top predators (e.g., snapper, barracuda, and grouper) can accumulate even higher CTX levels, posing a risk to human health [46,49]. Those toxin-equivalent concentrations must be considered carefully, however, because the venoms produced by lionfish itself can potentially interfere with the toxicity bioassays commonly used to quantify CTX. To avoid false-positive results on CTX tests, cooking the fillets of lionfish is recommended before testing for the presence of ciguatoxin. This procedure denatures the scorpaenitoxins, leaving only CTX-related toxicity if present [50]. Regardless, at least one case of CP following the consumption of lionfish has been already confirmed [51].

Considering the ecological relevance of *P. volitans* associated with its potential threats as an invasive species and emerging CP vector, this species was selected in the present study to study the transfer of CTX from a prey to a carnivorous reef fish predator. The potential effects of CTXs, as well as their transfer, accumulation, and elimination were investigated during a long-term (12-week) laboratory feeding experiment using naturally CTX-contaminated steephead parrotfish, *Chlorurus microrhinos* (formerly *Scarus gibbus*), fillets as source of toxins. The presence and concentration of CTXs were evaluated in liver and muscle tissues of juvenile *P. volitans* during both toxin uptake and depuration phases, using distinct analytical methods such as radioligand-receptor binding assay (r-RBA), neuroblastoma cell-based assay (CBA-N2a), and liquid chromatography coupled to tandem mass spectrometry (LC-MS/MS).

## 2. Results and Discussion

### 2.1. Experimental Setting and Fish Behavior

**Figure 1:**
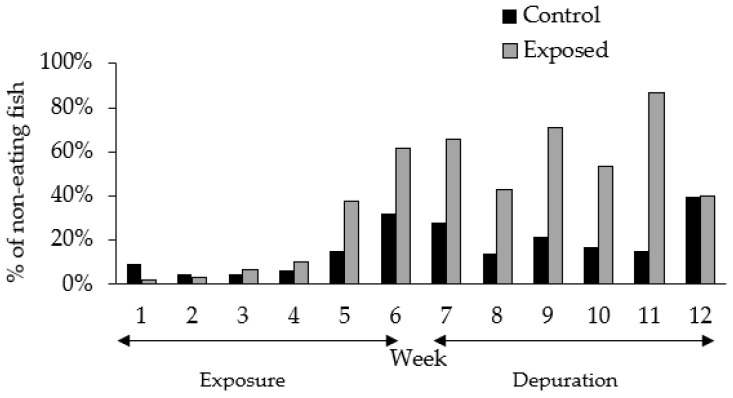
Proportion of control and CTX-exposed lionfish rejecting the food in relation to the number of remaining individuals at a given time during both the exposure and depuration periods.

The experimental feeding encompassed a 12-week period, including 6 weeks of exposure to CTX and 6 weeks of depuration. A gradual decrease in feeding activity was observed over the course of the experiment in both CTX exposed and control fish, although the propensity was greater among exposed fish (Figure 1). Increased food rejection episodes were indeed registered, mainly after the fifth week of experiment, during the late exposure period. The proportion of non-eating fish was 62% in the group exposed to CTX and 32% in the control group at late exposure phase (Figure 1). Such changes in feeding behavior were not observed in previous experiments using herbivorous fish (*Naso brevirostris*) or carnivorous groupers (*Epinephelus coioides*) exposed to diets containing 0.4 ng CTX3C equiv. g^−1^ over 16 weeks and ~1.0 ng CTXs equiv. g^−1^ day^−1^ (mean of CTX1B, 52-*epi*-54-deoxyCTX1B, 54-deoxyCTX1B quantifications) over 30 days of exposure, respectively [27,28]. However, the food rejection episodes observed during the present experiment did not seem to affect the CTX uptake and accumulation, since toxin levels were, in fact, detected in fish liver at the final exposure stage due to prolonged feeding (4–5 weeks) on CTX-containing parrotfish flesh (Table 1).

Although laboratory conditions appeared to be optimal and constant throughout the experiment, unexpected deaths were registered among both control and exposed lionfish (15 out of 48 individuals in total). Mortality also affected individuals belonging to a separate batch received after the beginning of the experiment, maintained under similar conditions in the facility for future experiments. The specimens used in this study may have become more sensitive and prone to diseases such as bacterial infections when transferred to a laboratory setting. Consequently, these unexpected fish mortalities led to the loss of experimental replicates. Despite the reduction of replicates and considering that (i) fish showed no signs of suffering during the experiment, (ii) CTX-contaminated matrices were difficult to obtain, and (iii) published information of CTX trophic transfer under controlled conditions are still very limited, we decided to maintain the experimental protocol. Diet comparisons (i.e., exposed vs. control group) were thus achieved using randomly sampled individual fish as replicates.

Increased food rejection episodes may have contributed to the fish deaths observed during our experiment, although this could not be confirmed. Indiscriminate, sporadic mortality episodes registered during the experiment seemed unrelated to the exposure to CTX. Out of the 32 CTX-dosed fish, 11 fish died, mostly on week 7 (after 1 week of depuration). Similarly, 4 out of 16 control fish died, but in this case, most deaths were registered on week 12, at the end of the experiment. The cumulative proportion of fish mortalities in the exposed group (36%) was similar to that of the control (28%).

During the entire experiment, exposed lionfish exhibited no clear signs of acute intoxication, such as erratic swimming, rapid gill movement, loss of equilibrium, and fin paralysis, as described in CTX-exposed mullet *Mugil cephalus* [25]. Likewise, in natural environments, CTX-contaminated fishes, including lionfish in the Caribbean region [49], as well as snapper and grouper on Pacific coral reefs [42], have been found to exhibit no signs of intoxication. Thus, coral reef fish species facing repeated exposure to CTXs in the natural environment may have developed resistance mechanisms to these compounds, perhaps binding them to particular soluble proteins in the skeletal muscle [52,53], or storing them in compartments where they would be biologically unavailable.

### 2.2. Toxin Levels in the Food and Fish Dosing

The average CTX content in the naturally contaminated parrotfish (*Chlorurus microrhinos*) fillet homogenate (toxic food) was estimated as 2.27 ± 0.4 ng CTX3C equiv. g^−1^ (coefficient of variation, CV = 17.6%) by r-RBA, with no significant difference (*p* < 0.05) among the eight subsamples tested. Analysis by CBA-N2a confirmed the presence of CTXs in the CTX-contaminated food, estimated at a concentration of 0.70 ± 0.02 ng CTX3C equiv. g^−1^ (*n* = 3) with CV of 2.8% (Supplementary Materials, Figure S1). Unfortunately, the toxin levels in parrotfish flesh were too close to the LOD and LOQ values by LC-MS/MS (up to 1.5 ng CTX3C equiv. g^−1^ and 4.7 ng CTX3C equiv. g^−1^ of flesh, respectively), to allow unambiguous determination of CTXs using this technique. As expected, the CTX-free food (control) exhibited no activity by r-RBA analysis in our experimental conditions.

Given the toxin content in the CTX-contaminated food and the daily ration of 0.05 g food per g of fish, individuals from the CTX-exposed group received a dose equivalent to 0.11 ng CTX3C equiv. g^−1^ d^−1^ according to r-RBA estimations, or 0.035 ng CTX3C equiv. g^−1^ d^−1^ by CBA-N2a quantification. The dose administrated herein can be representative of an exposure that can eventually occur in the reef environment (i.e., environmentally relevant dose), as high levels of CTXs (up to 10.7 ng CTX3C equiv. g^−1^) have been reported in parrotfish (*C. microrhinos*) sampled from French Polynesia islands [39].

Considering the average amount of food supplied and the toxin content in the experimental diet, the total toxin ingested per lionfish (initial body weight: 15.8 ± 9.6 g; *n* = 32) was estimated by r-RBA as 1.74 ng CTX3C equiv. day^−1^, totalizing 52.2 ng CTX3C equiv. and 71.3 ng CTX3C equiv. over 30 and 41 days, respectively, assuming that the fish accept all food portions offered. Based on the CBA-N2a analysis, each exposed lionfish may have ingested, on average, a 3.2-fold lower dose.

### 2.3. Toxin Levels in Fish Tissues

CTX were detectable by r-RBA in the liver of fish, yet not consistently. When quantified by this assay, toxin concentrations in the livers of exposed lionfish reached 9.4 ng CTX3C equiv. g^−1^ at day 41 of exposure to CTX-contaminated food. The highest toxin concentration (9.77 ng CTX3C equiv. g^−1^) was measured at late depuration phase (Table 1). As expected, CTX was not detected in the livers of control fish using either r-RBA (LOD: 0.75 ng CTX3C equiv. g^−1^) or LC-MS/MS (LOD: up to 1.14 CTX3C equiv. g^−1^ of liver). In fact, this latter analytical method proved to be the least sensitive one. Unfortunately, the limited sample volumes remaining from other technique, as well as the relatively low concentrations of individual CTX compounds present in our samples, together with the matrix effect, did not allow us to make any further clarification on the toxin profile of CTX-exposed fish. In French Polynesia, CTX composition of steephead parrotfish *C. microrhinos* typically includes CTX3C type compounds (71.1% as CTX3C and its M-seco form) and, to a lesser extent, CTX4A type (28.8% as CTX4A and its M-seco form) [33,54]. However, CTX profiles in fish are species-specific and can differ from regional variations [55,56]. The typical toxin profile reported in *Gambierdiscus* species found in Pacific coral reef is also mostly composed of the less polar CTXs (48% CTX3C, 34% 49-epiCTX3C and 13% CTX4A) [33].

Following CTX absorption, toxin metabolization in fish may affect the depuration and elimination process [37,55]. Of note, as suggested in the present study for lionfish, low CTX depuration rates were also reported in other carnivorous fishes, including experimentally exposed orange-spotted grouper (*Epinephelus coioides*) [28], as well as wild-caught moray eels *Gymnothorax javanicus* (*Lycodontis javanicus*) [57] and red snapper (*Lutjanus bohar*) [58]. For the latter species, the fish would require up to 30 months to completely eliminate the acquired toxin load. Moreover, the livers of contaminated orange-spotted groupers showed faster elimination rates for CTX1B, 52-*epi*-54-deoxyCTX1B, and 54-deoxyCTX1B, which are already highly metabolized toxins, compared to other tissues such as skin, gills, and muscles, indicating different CTX elimination rates among tissues of exposed fish over 30 days [28].

**Figure 2:**
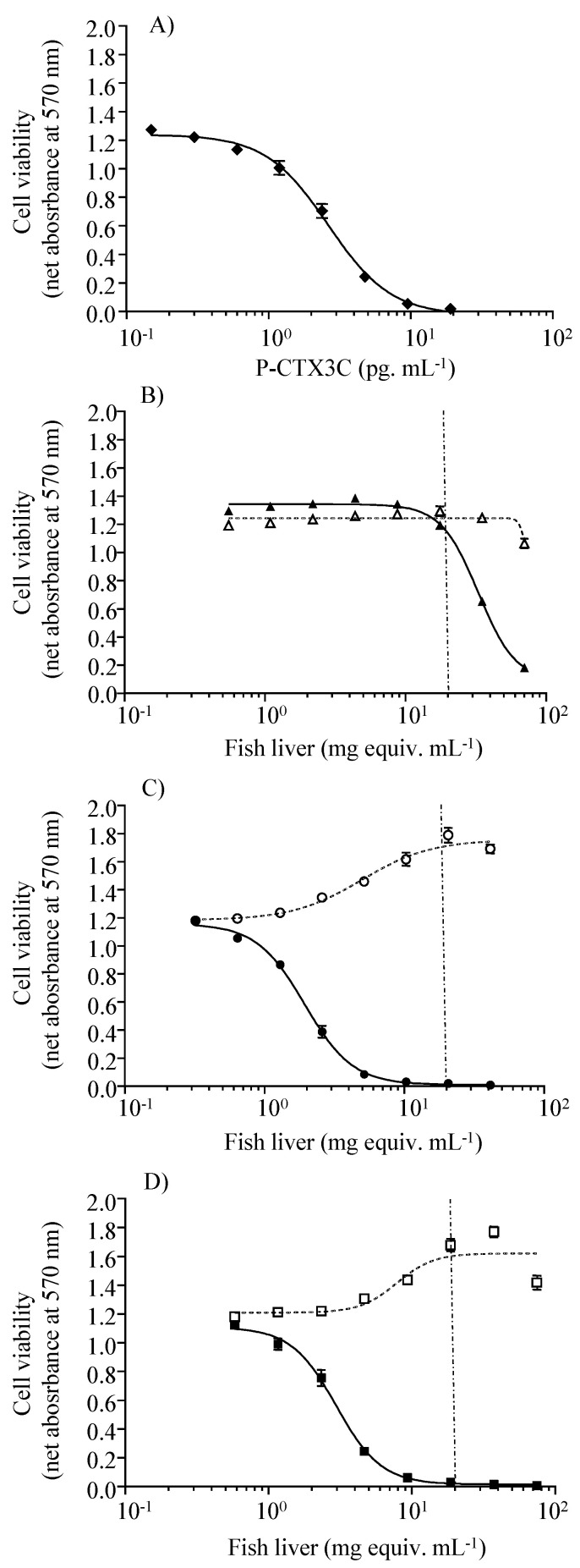
Quantification of CTX in fish liver during the exposure phase of the experiment. Dose-response curves of N2a cells when exposed to increasing concentrations of fish liver extracts, obtained from the exposure phase of the experiment, in OV^−^ (open symbols) and OV^+^ (solid symbols) conditions; (**A**) CTX3C (◆), (**B**) non-exposed control fish at day 42 (pool of 3 specimens) (∆/▲), (**C**) exposed fish at day 30 (pool of 4 specimens) (○/●), and (**D**) exposed fish at day 41 (pool of 5 specimens) (□/■). Data represent the mean ± SD of 1 assay, with each point run in triplicate. The dotted vertical line corresponds to the maximum concentration of liver tissue (MCE) that does not induce a matrix effect on the assay, which was estimated at 20 mg mL^−1^ of fish liver extracts.

**Figure 3:**
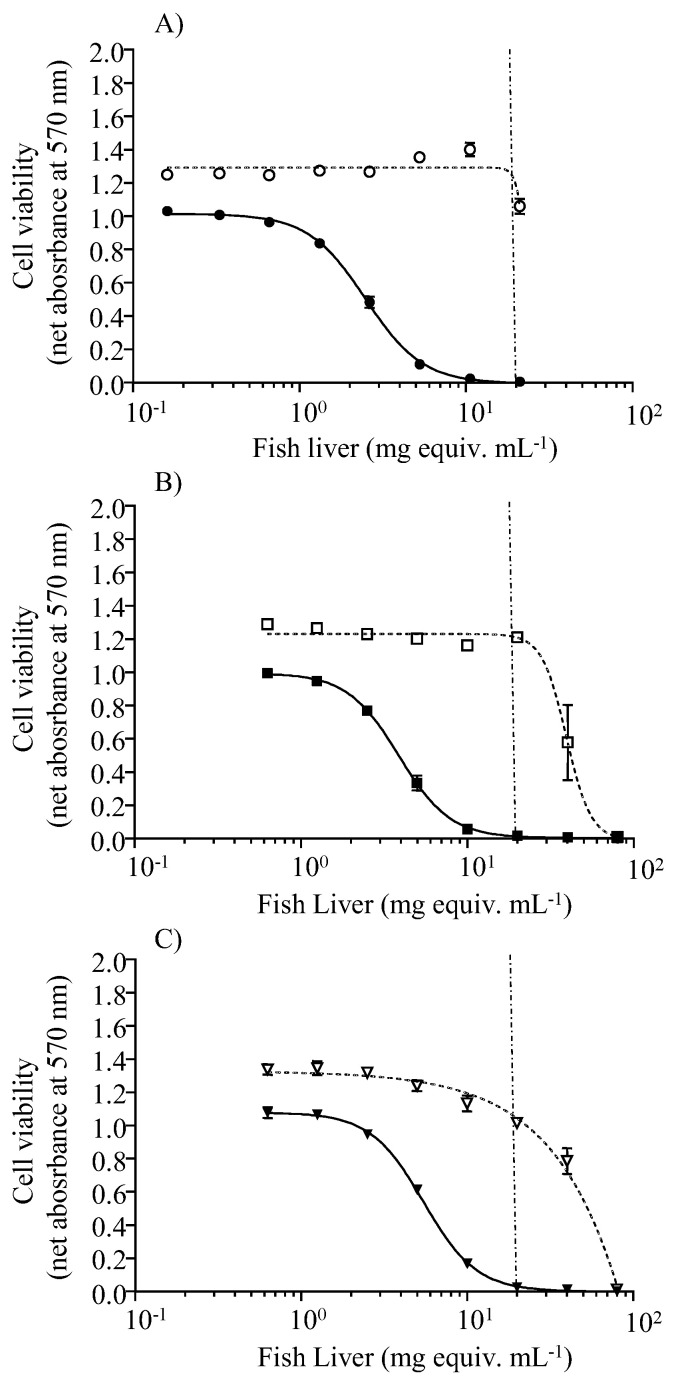
Quantification of CTX in fish liver during the depuration phase of the experiment. Dose-response curves of N2a cells when exposed to increasing concentrations of pooled fish liver extracts, obtained from the depuration stage of the experiment, in OV^−^ (open symbols) and OV^+^ (solid symbols); (**A**) 8 days of depuration (pool of 3 specimens) (○/●), (**B**) 29 days of depuration (pool of three specimens) (□/■), and (**C**) 43 days of depuration (pool of two specimens) (∆/▲). Data represent the mean ± SD of one assay, with each point run in triplicate. The dotted vertical line corresponds to the maximum concentration of liver tissue (MCE) that does not induce the matrix effect, which was established at 20 mg mL^−1^ of fish liver extracts.

Pooled liver extracts from control fish caused no cytotoxic effects on N2a cells in either OV^−^ or OV^+^ conditions throughout the experiment. Conversely, sigmoidal dose-response curves with a negative slope were obtained under the OV^+^ condition for liver extracts from fish sampled after 30 and 41 days of exposure to toxic food (Figure 2), and from those sampled after a depuration period of 8, 29, and 43 days (Figure 3). Based on the CTX-like composite toxicity using the CBA-N2a results, toxin concentrations in pooled lionfish livers were estimated as 1.36 ng CTX3C equiv. g^−1^ and 0.89 ng CTX3C equiv. g^−1^ after 30 and 41 days of exposure to toxin-containing food (herbivore *C. microrhinos* parrotfish fillets). Toxin concentration values decreased gradually over the depuration period, reaching 0.81 ng CTX3C equiv. g^−1^, 0.52 ng CTX3C equiv. g^−1^, and 0.37 ng CTX3C equiv. g^−1^ in pooled extracts from 8, 29, and 43 days of depuration on non-toxic food, respectively (Table 2). Considering the decrease in CTX-like cytotoxicity corresponding to a 0.44 ng CTX3C equiv. g^−1^ difference between days 8 and 43 of depuration (see Table 2), it would take an additional 35-day depuration period for lionfish livers to reach the CBA-N2a LOD of 0.06 ng CTX3C equiv. g^−1^. The kinetics of CTX elimination in lionfish liver, however, should be examined with caution, considering: (i) the limited number of time points for sampling during depuration stage (8, 23, and 43 days after last exposure), (ii) the use of pooled samples in CBA-N2a due to an insufficient amount of remaining individual tissues, and (iii) the limited amount of pooled tissues available, which did not allow for replication of the quantification of CTX in livers by CBA-N2a.

Livers of lionfish belonging to the CTX-exposed group weighed, on average, 0.64 g (3.4% of the total fish body weight), resulting in a toxin burden of up to 4.53 ng CTX3C equiv. and 6.04 ng CTX3C equiv. in this organ after 30 and 41 days of exposure, respectively (based on the r-RBA results). When referring to the CBA-N2a results, the average toxin burden in pooled livers ranged from 0.87 ng CTX3C equiv. to 1.90 ng CTX3C equiv. after 30 and 41 days of exposure, respectively. This represents a retention of 8.6% (r-RBA) or 6.8% (CBA-N2a) of the ingested toxin burden at the end of the exposure period. Recent studies have reported that carnivorous grouper (*Epinephelus coioides*) fish, fed over 30 days, demonstrated a toxin burden of ~1 ng CTX1B g^−1^ d^−1^—a greater toxin dose than that used in our experiment (0.11 ng CTX3C equiv. g^−1^ d^−1^ or 0.035 ng CTX3C equiv. g^−1^ d^−1^ from r-RBA or CBA-N2a analysis)—and accumulated up to 25% of the ingested toxin load in the livers and 10% in muscles [28]. In contrast, after 16 weeks of dietary exposure to 0.4 ng CTX3C equiv. g^−1^ d^−1^, juvenile *Naso brevirostris* accumulated ~2% of the exposed CTX3C amounts in muscular tissue [27].

In the present study, toxin concentrations in the muscles of lionfish belonging to the dosed group were always below the limit of detection, as determined by r-RBA. The sample amounts remaining from this first analysis were deemed insufficient for a supplementary evaluation by CBA-N2a. In natural environments, lionfish muscles were found to contain relatively high CTX levels (≥0.24 ng C-CTX1 equiv. g^−1^), indicating the potential of this species to cause human poisoning events [46,51]. When experimentally exposed to four-fold higher weight-specific daily doses of CTXs than those administered in our experiment, the herbivorous coral reef fish *Naso brevirostris* accumulated up to 3.24 ± 0.59 ng CTX3C equiv. g^−1^ in muscular tissues [27]. This value is within the range usually found among naturally contaminated fish in French Polynesia [41]. Moreover, in CTX-exposed *N. brevirostris*, the total amount of toxins in muscles increased linearly over 16 weeks of toxin exposure [27]. The muscles of *N. brevirostris* quickly eliminated the incorporated ciguatoxins, contrary to what has been observed for mullets (*Mugil cephalus*) after nine toxic feedings with gel food containing *Gambierdiscus* cells [25,27]. Finally, muscular tissues of groupers (*Epinephelus coioides*) incorporated the equivalent of 0.34 ng CTX1B g^−1^ d^−1^ after 30 days of exposure to 1 ng CTX1B g^−1^ d^−1^ [28]. In the Caribbean region, the highest C-CTX1 levels detected in the muscles of wild-caught fish were 0.24 ng g^−1^ in grey snapper (*Lutjanus griseus*), 0.3 ng g^−1^ C-CTX1 equivalents in lionfish (*P. volitans*), 0.9 ng g^−1^ in grouper (Serranidae), 13.8 ng g^−1^ in black jack (*Caranx lugubris*), and 49 ng g^−1^ in barracuda (*Sphyraena barracuda*) [49,59,60,61].

Despite the loss of replication and the need for a longer period of experimentation, our results indicate CTX transfer from contaminated prey to carnivorous *P. volitans*. Toxins concentrated into lionfish livers, while their transport to the muscles could not be reliably assessed. In other fish species, CTXs appear to be primarily accumulated in the liver, being secondarily distributed to the muscles and other tissues as the concentrations increase [28].

## 3. Conclusions

The accumulation of CTX in the livers of juvenile lionfish *Pterois volitans* became evident after 5 weeks of feeding toxin-contained parrotfish *Chlorurus microrhinos* fillets. The toxin levels in the muscles of lionfish were always below the limits of detection, as determined by r-RBA, suggesting potential differential tissue distribution over the experiment. However, this could not be confirmed by a more sensitive method, such as CBA-N2a due to the insufficient amount of flesh. Lionfish retained detectable toxin levels in their livers at 43 days after the last exposure, indicating a slow elimination process. No acute intoxication signs were observed throughout the experiment, suggesting the resistance of *P. volitans* to CTXs.

## 4. Materials and Methods

### 4.1. Fish Acclimation and Maintenance

Juvenile lionfish, *Pterois volitans* (Scorpaenidae), originating from Bali, Indonesia, were acquired from a supplier (Tropic Nguyen, France). The juvenile lionfish were selected based on maintenance and individual replication needs considering the laboratory setting and aquarium size. Upon reception, the lionfish were acclimated for approximately 2 weeks in a 2000 L open-circuit tank. The thank was filled with flowing 1 μm filtered seawater at 400 L h^−1^, 25 ± 0.5 °C, pH 8.1 ± 0.1, and a salinity of 39, and maintained under a 12:12 h (light:dark) cycle and permanent aeration. Fish were fed once a day during acclimation in amounts higher than needed for maintenance (equivalent to 5% of their body weight). The food consisted initially of living prey (guppy or seabream). Then, inert frozen food (*Antherina boyeri* and krill) was gradually incorporated into the diet before the final transition to the frozen, CTX-containing parrotfish (*Chlorurus microrhinos*) fillet.

### 4.2. Preparation of the Experimental Diets

The toxic material used for the feeding experiments consisted of naturally CTX-contaminated fillets of the steephead parrotfish *Chlorurus microrhinos* collected from Moruroa Atoll (Tuamotu Archipelago, French Polynesia). Parrotfish were selected based on the CTX levels in their fillets. Then, 7.8 kg (wet weight) of selected fillet were homogenized using an industrial mixer to obtain a toxic, homogenous fish fillet matrix. Toxicity of the prepared food was evaluated by means of cytotoxicity using CBA-N2a and binding affinity using r-RBA composed of 3 × 10 g and 8 × 5 g aliquots of the dried homogenate, respectively.

Different food preparations using Gelly Belly™ (Gel Food, Florida Aqua Farms, Inc., Dade City, FL, USA) and frozen food were tested. Gelly Belly™ consists of a gelatin-based food mixed with microalgae, seaweeds, fish, and krill meal, with added vitamins and minerals. The most appropriate food presentation for lionfish proved to be homogenized cube-shaped frozen fillets. Frozen food dices were individually offered to lionfish using forceps, which were instantaneously caught and ingested without significant particle loss.

The food provided to the individuals in the control group—and to all remaining lionfish during the depuration phase—was composed of homogenized fillet of farmed seabream (*Sparus auratus*; ~120–140 g total body weight), originated from Turkey and supplied by Relais d’Or (France). After homogenization, seabream fillets were fashioned into cubes, placed in plastic bags, and kept at −18 °C until being offered to the carnivorous fish as frozen cubes. A sample of seabream fillets was collected and further tested for CTX presence.

### 4.3. Experimental Design

**Figure 4:**
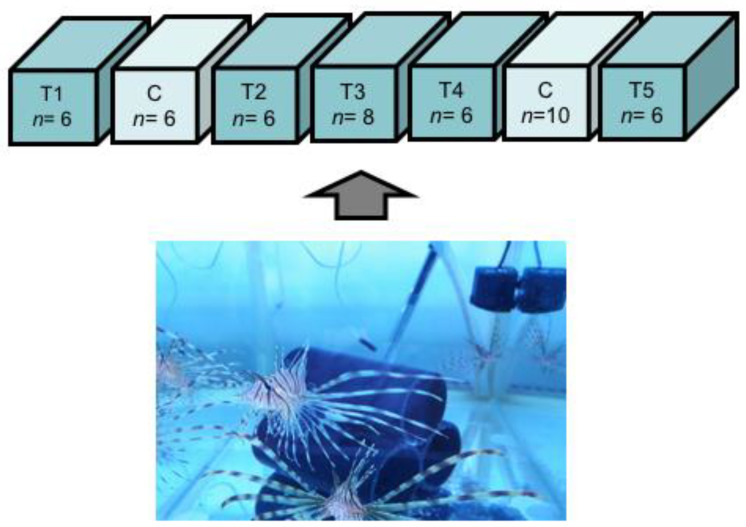
Design of the experiment, with 5 tanks initially containing 6 or 8 fish (*n*) assigned to the CTX-exposed treatment (T) and 2 tanks for control (C) containing 6 fish and 10 fish (*n*). The image represents a picture of the juvenile lionfish (*Pterois volitans*) individuals and their shelters made of dark tubes in one of the experimental tanks.

After acclimation, *P. volitans* individuals (*n* = 48; 5.05–34.0 g initial wet weight) were distributed among seven aquaria, allocating six fish per tank, except in two tanks where ten and eight fish were placed due to the smaller size of some individuals (~5 g) (Figure 4). This step aimed at obtaining similar fish sizes and total living biomass (15.2 g ± 9.52, mean ± SE) across the experimental tanks. Tanks were randomly assigned to either toxin-containing (5 tanks) or control diet (2 tanks), each containing 100 L of filtered seawater maintained under constant aeration and flow (100 L h^−1^), at the same conditions of temperature, salinity, and pH used during the acclimation phase. To reduce animal stress, three tubes of 10 cm × 20 cm (D × L), assembled as a pyramidal structure, were placed inside the aquaria to provide shelter for the fish (Figure 4). Finally, the aquarium walls were covered with opaque plastic coating to limit potential stress caused by the human presence in the laboratory. Standards of animal welfare were rigorously maintained throughout the experiment. All procedures were carefully conducted to minimize handling and reduce physiological stress.

After 6 weeks of exposure to either the toxin-containing or control diet, fish entered the depuration stage, during which they received non-contaminated frozen food (homogenized seabream fillets) for an additional 6-week period. Fish feedings were carefully conducted throughout the experiment, assuring quick capture (usually <5 min) and no significant particle rejection. Some individuals in the control group were maintained over the entire duration of the experiment (12 weeks) and served as reference for possible visual behavior alterations and/or intoxication symptoms relative to dosed fish. The behavioral pattern was observed daily in all tanks during the feeding period throughout the 12-week experiment. During the exposure and depuration phases, the number of deaths was computed in relation to the total number of fishes at the beginning of the experiment, while the percentage of fish rejecting the toxic food considered the average number of individuals remaining in each experimental condition.

#### Feeding Experiment

During the experiment, all lionfish individuals were fed in a constant proportion to 4–5% of their body weight per day, as recommended for juvenile fish. The amount of food supplied was adjusted weekly based on the average fish weight for each tank. The lionfish were individually fed, always in the afternoon (2:00 p.m.), 5 days a week. Individuals were observed over 30 min to assure complete food ingestion after every feeding procedure. At the end of this time interval, any rejected food was removed from the tank during the daily cleaning procedure. Lionfish usually consumed the entire portion of toxic food offered in less than 15 min. In addition, the tanks were connected to an open system under a constant seawater flow. Thus, any residual toxin eventually released from the food—or excreted by the fish—was quickly eliminated from the tanks. Therefore, the source of CTX to the exposed fish was considered limited to the food pathway.

After 30 days of exposure, four CTX-exposed and three control lionfish were randomly collected from the control tanks. Likewise, between two and five individuals from the exposed group were sampled each time, at the end of the exposure period (41 days) and after 8, 29, and 43 days of the following depuration stage. Finally, three additional control individuals were collected after 8, 29, and 43 days of the depuration stage (Table 3). Fish euthanasia was achieved using an overdose of eugenol, and the death was confirmed by the absence of respiratory movements [62]. Lionfish were dissected immediately following death to ensure the integrity of collected tissues. For toxin analysis, muscle and liver samples were individually weighed (wet weight) and stored in plastic tubes at −18 °C.

### 4.4. Toxin Determination in Food and Exposed Fish

#### 4.4.1. Sample Extraction

The extraction of CTXs from the muscle and liver samples followed the procedures described in previous studies [27,63,64]. Briefly, each tissue sample from individual lionfish was extracted as a whole when the tissues weighed <4 g, or partially, in 4 g aliquots after homogenization with T-25 digital Ultra Turrax (IKA Works, Staufen, Germany). Tissue samples were then cooked in Falcon tubes in a water bath at 70 °C for 15 min, homogenized in acetone (3 mL g^−1^ tissue) using a sonication probe (Branson digital probe cell breaker) for 2 min at 30% duty, and centrifuged for 3 min at 1400× *g*. The supernatant was recovered from the tubes, and the tissue pellet was homogenized twice again in acetone, as previously described. After three successive extraction steps, the supernatant fractions were combined and evaporated under nitrogen gas flow (Turbovap) in a water bath at 60 °C. Dried extracts were resuspended in 5 mL of aqueous methanol (MeOH/H_2_O 90:10), and the lipids were removed by solvent–solvent separation (three times) after the addition of an equal volume of *n*-hexane. The 1:1 solvent–solvent fraction was separated (3×) into an aqueous phase (MeOH/H_2_O 60:40) and dichloromethane (DCM). Finally, the organic phase (DCM), containing the CTXs, was evaporated with nitrogen, resuspended in pure MeOH to 10 g tissue equivalent (TE) mL^−1^, and stored at −18 °C until further analysis. Frozen homogenized fillet cubes of parrotfish *Chlorurus microrhinos* (toxic food) and seabream (control food) were extracted following the same procedure described for lionfish tissues, adjusting the sonication time to 20 min to ensure complete fish cell lysis.

#### 4.4.2. Toxin Analysis

##### Radioligand-Receptor Binding Assay (r-RBA)

The presence and quantification of CTXs in parrotfish (*C. microrhinos*) fillet homogenates (toxic food) and in selected extracts of fish muscle and liver were first determined using the r-RBA [65]. This detection method is based on the binding competition between CTXs from the sample and a radiolabeled brevetoxin (tritiated PbTx-3) for their common receptor—on the voltage gated sodium channel (Na_v_)—using a porcine brain homogenate (Sigma Aldrich, St. Louis, MO, USA) membrane preparation [11,65].

The assay was performed on a microplate according to the method reported by the authors of [63], modified according to previous studies [27,64,65]. First, 35 μL of phosphate-buffered saline (PBS-Tween^®^) with bovine serum albumin (BSA) (1 g L^−1^) was added to each well of a 96-well filtration microplate (MultiScreen HTS FB Filter Plate MSFBN6B50, Millipore) to moisturize the membrane filter. Subsequently, 35 µL of either the standard CTX3C provided in dried form by Wako-Pure Chemicals, Osaka, Japan (2.85 × 10^−9^ to 1.92 × 10^−12^ M), a solution of PbTx-3 (Latoxan, Rosam, France) (1.8 × 10^−8^ M) used as an internal assay Quality Control (QC) (3 × 10^−9^ M in assay), or the diluted samples were added to each corresponding well, after vortex-mixing and sonication. Likewise, only one concentration of fish sample was tested, i.e., 0.6 g tissue equiv. mL^−1^ for the parrotfish toxic food, and 0.6 g tissue equiv. mL^−1^ and 0.12 g tissue equiv. mL^−1^ for lionfish muscle and liver, respectively. Each sample was tested in duplicate wells in one experiment. Then, 35 µL of the working solution of [^3^H] PbTx-3 (1 nM assay concentration) and 195 µL of the diluted brain membrane homogenate (0.8 mg protein mL^−1^) were sequentially added to all wells. After incubation for 1 h at 4 °C, each well was washed (3×) with 200 µL of ice-cold phosphate-buffered saline solution (PBST) and filtered using a MultiScreen HTS vacuum collector system (Milipore, Billerica, Massachusetts, USA) to remove the excess radiotracer [^3^H] PbTx-3. Only the membrane receptor-bound toxin molecules were retained in the filter at this stage. After filtration, the microplate was placed on a counting cassette (Perkin-Elmer rigid 96 plate 14105), with 50 µL of liquid scintillant (Optiphase, Perkin-Elmer, USA) per well. Finally, the plate was incubated in the dark at room temperature for 2 h prior to quantification of the radioactivity in a beta-microplate counter (MicroBeta^2^, Perkin-Elmer, Waltham, MA, USA).

##### Liquid Chromatography Coupled with Tandem Mass Spectrometry (LC-MS/MS)

Analysis by LC-MS/MS was performed on liver samples of lionfish and in CTX-contaminated parrotfish *Chlorurus microrhinos* homogenates to confirm the presence of CTXs in the extracts. Aliquots of the extracts remaining from the r-RBA analysis (10–100 µL) were dried off with nitrogen gas and resuspended with the following volume of 90% aqueous MeOH: 200 µL for lionfish liver samples and 500 µL for parrotfish homogenate, yielding a final concentration of 0.5–2.6 g liver equiv. mL^−1^ and 0.4–1.1 g flesh equiv. mL^−1^, respectively. Different dilutions reflected the amount of matrix available in each case. Toxin determination was performed on a UHPLC system (UFLC Nexera, SHIMADZU, Japan) coupled to a hybrid triple quadrupole-linear ion-trap API4000 Qtrap mass spectrometer (ABSciex^®^, Framingham, MA, USA), equipped with a TurboV^®^ electrospray ionization source.

Eluents consisted of deionized water (A) and 95% acetonitrile (B), both containing 2 mM ammonium formate and 50 mM formic acid. The following linear elution gradient was run at 0.4 mL min^−1^ through a Zorbax Eclipse Plus C18 column (50 × 2.1 mm, 1.8 μm, 95 Å; Agilent Technologies, Santa Clara, CA, USA), maintained at 40 °C: 78 to 88% B in 10 min, held at 88% B for 4 min, decreased back to 78% in 1 min, and held during 5 min to equilibrate. Samples were kept at 4 °C during the analysis, and 5 μL aliquots were injected into the system. Scheduled MRM scanning (90 s detection window; 2 s target scan time) was applied in positive electron spray ionization (ESI+) mode, using the following optimized parameters: curtain gas at 25 psi; ion spray at 5500 V; turbo gas temperature at 300 °C; gas 1 and 2 at 40 and 60 psi, respectively; declustering potential at 105 V; and entrance potential at 10 V. A list of MRM transitions (*m/z*) scanned in ESI+ for the detection of CTX-like compounds is given in Table 4, along with the respective collision energy (CE) and targeted retention time values. Instrument control, data processing, and analysis were conducted using Analyst software 1.6.2 (SCIEX, CA, USA). Due to the lack of standards, calibration curves of CTX3C (Wako, Japan) were applied to calculate the concentration of every compound detected, assuming an equivalent molar response for the other analogs.

##### Cell Based Assay on Neuroblastoma (CBA-N2a)

The neuroblastoma (N2a) CCL 131 cell line (ATCC) was used in this study, and the CBA-N2a was performed following the protocol described by the authors of [66]. Briefly, the 60 inner wells of several 96-well microplates were seeded with 200 µL of a 5% FBS culture medium at an initial cell density of 50,000 ± 10,000 cells well^−1^ (exponential growth phase) and left to grow for 26 h in an incubator at 37 °C and 5% CO_2_. Among the several microplates run in parallel, one microplate served as the Reference Cell Viability (RCV) control to establish the initial cell viability of N2a cells using an MTT assay [66]. For each microplate, the mean absorbance of DMSO control (12 outer wells filled with DMSO only) was subtracted from each raw absorbance value (60 inner wells containing N2a cells), and all viability data were expressed in net absorbance data.

The remaining microplates were treated as follows. The growth medium was renewed by the addition of 200 µL of 2% FBS culture medium with 90 µM of ouabain (O) and 9 µM of veratridine (V) for non-destructive treatment in the OV^+^ condition (bottom half of the microplate). In the OV^−^ condition, the growth medium was renewed by the addition of 200 µL of 2% FBS culture medium (upper half of the microplate). Appropriate controls in both OV conditions, namely COV^−^ and COV^+^, were established by adding 10 µL of 2% FBS culture medium to verify the final cell viability and the non-cytotoxicity of the OV treatment, respectively, in the absence of CTXs. The implementation of additional quality check controls (QC) to both OV^-^ and OV^+^ conditions, namely QCOV^−^ and QCOV^+^, was also undertaken to check for the specific effect of voltage gated sodium channel (VGSC) activators. Basically, 10 µL of PbTx3 (Latoxan, France) at 0.1 µg mL^−1^ were added in triplicate wells in both OV conditions to reach a final concentration of 4760 pg PbTx3 mL^−1^ in the wells. As CTX3C was tested in parallel with the fish samples, eight-point 1:2 serial dilutions of the CTX3C standard (Institut Louis Malardé) and sample stock solutions were prepared (100 µL per concentration) using a U-bottom 96-well microtiter. Then, 10 µL of CTX3C concentrations were directly added in triplicate wells under the non-destructive OV^+^ condition (85.7/8.57 µM final concentrations) and not under OV- condition, since no cytotoxicity occurs in the absence of the O/V treatment [66]. The final concentrations of CTX3C tested ranged from 0.15 to 19.05 pg mL^−1^. The three parrotfish (toxic food) aliquots were tested with final concentrations ranging from 74 to 9524 ng mL^−1^ of dry extract (corresponding to 0.23–29.76 mg flesh equiv. mL^−1^). Each concentration was tested in triplicate wells in both OV conditions, in three independent microplates run the same day.

Liver extracts were further analyzed by CBA-N2a. To this end, all liver extracts prepared from individual fish collected from a single tank at the same sampling interval (days 30, 41 of the exposure phase and days 8, 29, and 43 of the depuration phase). Pooled samples ranged from 0.11–0.51 g fresh weight equivalent of fish liver. These samples were resuspended in a solution of 5 µL MeOH, 15 µL dimethyl sulfoxide (DMSO), and 2% fetal bovine serum (FBS) RPMI medium, providing concentrations of sample stock solutions ranging from 0.442 g fresh liver equiv. mL^−1^ to 1.567 g fresh liver equiv. mL^−1^, with no more than 10% of solvent used during the serially two-fold dilution procedure. As dry extract weights were not available for lionfish livers, fresh fish liver equivalents could only be considered to estimate the concentration ranges tested. Pools of lionfish liver from the exposure experiment were tested together in one CBA-N2a experiment. In the same way, pooled liver from the depuration phase were also tested together in another CBA-N2a experiment. For each CBA-N2a experiment, CTX3C was tested in parallel with the lionfish liver samples, each concentration tested in triplicate wells in both OV conditions. Full dose-response curves were used for accurate CTX quantification. These two CBA-N2a experiments could not be repeated due to the very small amount of extract available. In all microplates, peripheral wells received 200 µL of sterile distilled water, and microplates were left to incubate overnight for about 19 h prior to the determination of the final cell viability using the MTT assay with an incubation time of 45 min.

### 4.5. Data Analysis

#### 4.5.1. r-RBA

The r-RBA result of each extract was determined after the assay performance was confirmed by the Quality Control (QC), together with the additional parameters of the CTX3C standard curve (Supplementary Materials, Figure S2), such as the effective concentration inducing a 50% values effect (EC_50_), and the slope of the linear portion of the standard curve. Sample CTX quantification was only completed when the CPM measurement of a sample dilution fell on the linear part of the CTX3C standard curve and the relative standard deviation (rSD) of the triplicate CPM values was verified to be less than 30%. The Hill equation was used to convert CPM values into CTX concentrations.

Data analysis was performed in GraphPad Prism version 6.0 (San Diego, CA, USA), allowing the determination of the parameters of each CTX3C competition sigmoidal curve (using 4 parameters). The detection limit (LOD) and the limit of quantification (LOQ) of the CTX-like composite in fish muscle and liver samples were determined based on the relative standard deviation (rSD) of the top plateau (Bmax) of the dose-response curve according the method described by the authors of [64], following the equations: LOD = Bmax–3 * rSD; and LOQ = Bmax–10 * rSD. The values of LOD and LOQ were not estimated in this study. However, previous publications have reported LOQ values corresponding to 0.32 ng CTX3C equiv. g^−1^ [27] and 1.5 ng CTX3C equiv. g^−1^ [36] and LOD values of 0.75 ng CTX3C equiv. g^−1^ [64].

#### 4.5.2. LC-MS/MS

A calibration curve was calculated from the successive dilutions of a CTX3C standard solution (Wako, Tokyo, Japan) in MeOH at concentrations ranging from 12.5 ng mL^−1^ to 200 ng mL^−1^. The limit of detection (LOD) and limit of quantification (LOQ) were calculated statistically based on the following formulae: LOD = 3.3 * std/S; and LOQ = 10 * std/S, where “std” is the standard deviation and “S” the slope of the calibration curve composed of successive dilutions of the CTX3C reference material. The calculated values for LOD and LOQ corresponded to 0.57 ng CTX3C mL^−1^ and 1.74 ng CTX3C mL^−1^, respectively. This was equivalent, respectively, to 0.22–1.14 ng CTX3C mL^−1^, and 0.67–3.48 ng CTX3C g^−1^ of lionfish liver, and to 0.54–1.54 and 1.64–4.71 ng CTX3C g^−1^ of parrotfish *Chlorurus microrhinos* flesh.

#### 4.5.3. CBA-N2a

Net absorbance data were used to establish the full sigmoidal dose-response curves for CTX3C, lionfish liver extracts, and parrotfish *Chlorurus microrhinos* toxic food. The CTX3C and fish samples were tested in parallel in the same assay. The maximum concentration of the liver tissue (MCE) that did not induce unspecific mortality on N2a cells was determined in OV^−^ and OV^+^ conditions using the fish control sample from the exposure phase of the experiment. This concentration was further used to establish the concentration range of the fish liver samples to use for the assay in the depuration experiment. The maximum concentration of dry extract (MCE) was defined as 20 mg mL^−1^ from the fish liver control at 42 days. The limit of detection (LOD) and the limit of quantification (LOQ) of the CTX-like toxicity in the fish liver samples, expressed in ng CTX3C equiv. g^−1^ of fish liver, were determined according to the following equations: LOD = (EC_80_/MCE) and LOQ = (EC_50_/MCE), where EC_80_ and EC_50_ are the values obtained for CTX3C toxin standard, and were determined at 0.06 ± 0.01 and 0.12 ± 0.02 ng CTX3C equiv. g^−1^, respectively. The composite cytotoxicity in the fish liver samples, expressed in ng CTX3C equiv. g^−1^ of fish liver, was then estimated based on the (EC_50_ of CTX3C/EC_50_ of fish liver) equation.

## Figures and Tables

**Table 1:** Detection and quantification of ciguatoxins in individual lionfish livers during the experimental exposure and depuration periods by r-RBA analysis.

Periods of Experiment	Fish Individual	Days of Exposure/ Depuration	Fish Total Weight (g)	Liver Wet Weight (g) (% Contribution)	Toxin Concentration (ng CTX3C Equiv. g^−1^)
	1	30	20.3	0.53 (3%)	<LOD **
Exposure	2	30	16.9	0.45 (3%)	7.08
	3	30	10.0	0.30 (3%)	<LOD **
	4	30	5.56	0.08 (1.5%)	<LOD **
	5	41	21.0	0.74 (3.5%)	<LOD **
	6	41	14.4	0.33 (2%)	<LOD **
	7	41	17.6	0.39 (2%)	9.43
	8	41	14.4	0.40 (3%)	<LOD **
	9	41	9.08	0.22 (2%)	<LOD **
Depuration	10	8	19.4	0.65 (3%)	9.30
	11	8	15.0	0.61 (4%)	9.42
	12	29	16.7	0.55 (3%)	8.93
	13	29	8.72	0.02 (0.2%)	<LOD **
	14	29	9.81	0.11(1.1%)	<LOD **
	15	43	21.2	0.34 (2%)	9.77
	16	43	10.3	0.18 (2%)	<LOD **

** LOD = the limit of detection of the r-RBA was 0.75 ng CTX3C equiv. g^−1^.

**Table 2:** Estimation of the EC_50_ values and the CTX-like concentration of the pooled lionfish liver samples, as determined by the CBA-N2a analysis of the control (CTX-free food) and during exposure (CTX-contaminated food) and depuration (CTX-free food) periods of the experiment.

Periods of Experiment	Days of Exposure/ Depuration	Pooled Samples (*n*)	Fish Liver Weight Per Pool (g)	EC_50_ (mg Equiv. mL^−1^)	Toxin Concentration (ng CTX3C equiv. g^−1^)
Exposure	30	4	0.47	1.93	1.36
	41	5	0.19	2.97	0.89
Control	42	2	0.50	ND *	<LOD **
	8	1	0.51	ND *	<LOD **
Depuration	8	2	0.23	2.49	0.81
	29	3	0.13	3.88	0.52
	43	2	0.11	5.46	0.37

* ND = not determined as control samples gave no cytotoxicity at the MCE in both OV conditions. ** LOD = limit of detection of the CBA-N2a (0.06 ± 0.01 ng CTX3C equiv. g^−1^).

**Table 3:** Number of CTX-exposed juvenile *Pterois volitans* individuals (*n*) sampled after a given exposure or depuration period. In addition, fish fed non-toxic food (control) were sacrificed after 30 days of exposure and after 8, 29, and 43 days of depuration period (3 individuals at each time).

Periods of Experiment	Cumulative Number of Experimental Weeks	Days of Exposure/ Depuration	Cumulative Number of Feeding Episodes Toxic/Control Food	*n*
Exposure	5	30 days of exposure	22/0	4
	6	41 days of exposure	29/0	5
Depuration	7	8 days of depuration	29/6	3
	10	29 days of depuration	29/20	3
	12	43 days of depuration	29/30	2

**Table 4:** List of MRM transitions (*m/z*) used in ESI+ to detect CTXs by LC-MS/MS.

Compound	MRM Transitions (*m/z*)		Precursor Ion Species	Targeted Time (min)	CE (eV)
	Precursor Ion (Q1)	Product Ion (Q3)			
CTX3C or CTX3B	1040.6	1005.6	[M + NH4]^+^	11.2	30
	1023.6	1005.6	[M + H]^+^		20
	1023.6	125.1			50
M-*seco*-CTX3C	1041.6	1023.6	[M + H]^+^	4.7	20
	1041.6	1005.6			30
	1041.6	125.1			50
2-hydroxyCTX3C	1058.6	1005.6	[M + NH4]^+^	5.0	30
	1058.6	1023.6			20
	1058.6	125.1			50
51-hydroxyCTX3C	1056.6	1021.6	[M + NH4]^+^	6.3	30
	1039.6	1021.6	[M + H]^+^		20
	1039.6	1003.6			20
CTX4A or CTX4B	1078.6	1043.6	[M + NH4]^+^	12.8	30
	1061.6	1043.6	[M + H]^+^		20
	1061.6	125.1			50
54-deoxyCTX1B or 52-*epi*-54- deoxyCTX1B	1112.6	1077.6	[M + NH4]^+^	6.8	20
	1112.6	1059.6			30
	1112.6	95.1			90
CTX1B	1128.6	1093.6	[M + NH4]^+^	3.2	20
	1128.6	1075.6			30
	1128.6	95.1			90
C-CTX1 or C-CTX2	1141.4	1123.4	[M + H]^+^	4.4	30
	1123.4	1105.6	[M + H–H2O]^+^		30
	1123.4	1087.6			30
2,3-dihydro-2,3- dihydroxyCTX3C	1074.6	1039.6	[M + NH4]^+^	6.0	30
	1057.6	1039.6	[M + H]^+^		20
	1057.6	125.1			50
CTX3C analog 1	1040.6	1005.6	[M + NH4]^+^	10.0	30
	1023.6	1005.6	[M + H]^+^		20
	1023.5	125.1			50
CTX3C analog 2	1040.6	1005.6	[M + NH4]^+^	8.5	30
	1023.6	1005.6	[M + H]^+^		20
	1023.5	125.1			50
CTX3C analog 3	1040.6	1005.6	[M + NH4]^+^	7.5	30
	1023.6	1005.6	[M + H]^+^		20
	1023.5	125.1			50

## Data Availability

The data presented in this study are available in this article and Supplementary Materials.

## Supplementary Materials

The following are available online at https://www.mdpi.com/article/10.3390/toxins13080564/s1, Figure S1: Dose-response curves of N2a cells when exposed to increasing concentrations of parrotfish flesh extracts (toxic food) in OV^−^ (open symbols) and OV^+^ (solid symbols) conditions at 85.7/8.75 µM (final concentrations). Data represent the mean ± SD of each aliquot tested, with each point run in triplicate. Absorbance values were measured at 570 nm via the MTT assay, after a 45 min MTT incubation time. The initial cell viability was 1.054 ± 0.020 in the RCV control. The mean final cell viability was 0.927 ± 0.023 in the absence of O/V treatment (COV−), and 1.031 ± 0.029 in the presence of non-destructive O/V treatment (COV+), respectively. The dotted vertical line corresponds to the MCE established at 10,000 mg mL^−1^ of fish flesh extracts, avoiding non-specific cy-totoxicity in both conditions of OV treatments. The LOD and LOQ values in fish flesh were 0.03 ± 0.01 and 0.06 ± 0.02 ng CTX3C equiv. g^−1^ [66], Figure S2. The sigmoidal dose-response curve of r-RBA was used to quantify the concentration of the CTX in the fish liver and muscle during the experiment (GraphPad Software, Inc., La Jolla, CA, USA).
