# Supplementary material for: Experimental Evidence of Ciguatoxin Accumulation and Depuration in Carnivorous Lionfish
Source: Toxins (Basel). 2021 Aug 11;13(8):564. doi: 10.3390/toxins13080564 (PMC8402466; doi:10.3390/toxins13080564)
Supplement: Supplementary file 1 [file toxins-13-00564-s001.zip › toxins-1291001-supplementary DONE.pdf]

Supplementary Materials: Experimental Evidence of Ciguatoxin Accumulation and Depuration in Carnivorous Lionfish

Isabel do Prado Leite, Khalil Sdiri, Angus Taylor, Jérôme Viallon, Hela Ben Gharbia, Luiz Laureno Mafra Júnior, Peter Swarzenski, François Oberhaensli, Hélène Taiana Darius and Mireille Chinain, Marie-Yasmine Dechraoui Bottein

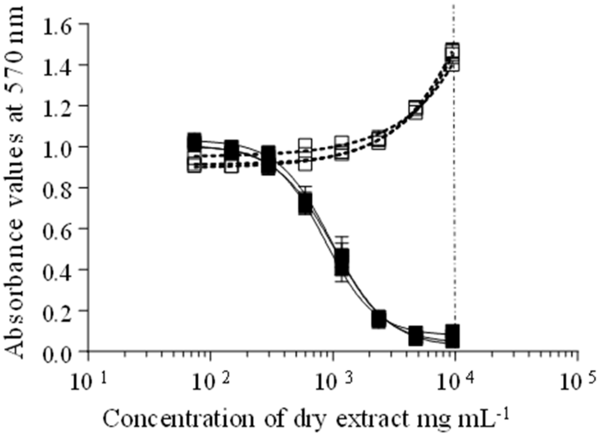

Figure S1. Dose-response curves of N2a cells when exposed to increasing concentrations of par-rotfish flesh extracts (toxic food) in OV− (open symbols) and OV+ (solid symbols) conditions at 85.7/8.75 μM (final concentrations). Data represent the mean ± SD of each aliquot tested, with each point run in triplicate. Absorbance values were measured at 570 nm via the MTT assay, after a 45 min MTT incubation time. The initial cell viability was 1.054 ± 0.020 in the RCV control. The mean final cell viability was 0.927 ± 0.023, in absence of O/V treatment (COV-), and 1.031 ± 0.029 in the presence of non-destructive O/V treatment (COV+), respectively. The dotted vertical line corre-sponds to the MCE established at 10 000 mg mL-1 of fish flesh extracts avoiding non-specific cy-totoxicity in both conditions of OV treatments. The LOD and LOQ values in fish flesh were 0.03 ± 0.01 and 0.06 ± 0.02 ng CTX3C equiv. g-1 [64].

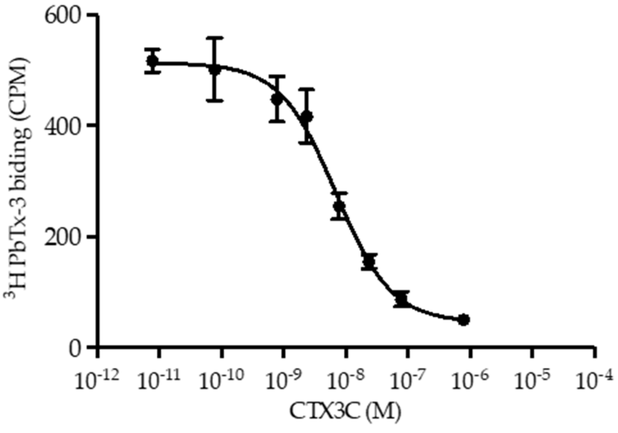

Figure S2. Sigmoidal dose-response curve of r-RBA used to quantify the concentration of the CTX in fish liver and muscle during the experiment (GraphPad Software, Inc., La Jolla, California, USA).
